# Anti-PD-1/PD-L1 for nasopharyngeal carcinoma: a comprehensive analysis of registered trials on ClinicalTrials.gov

**DOI:** 10.3389/fphar.2023.1212813

**Published:** 2023-11-13

**Authors:** Zelei Dai, Nian Li, Jun Wang, Chenfeng Tan, Yonggang Zhang, Lei Liu

**Affiliations:** ^1^ Division of Head and Neck Tumor Multimodality Treatment, Cancer Center, West China Hospital, Sichuan University, Chengdu, China; ^2^ Department of Medical Administration, West China Hospital, Sichuan University, Chengdu, China; ^3^ Department of Evidence Based Medicine and Clinical Epidemiology, West China Hospital, Sichuan University, Chengdu, China

**Keywords:** nasopharyngeal carcinoma, anti-PD-1/PD-L1 antibodies, clinical trial, treatment, comprehensive analysis

## Abstract

**Objective:** Clinical trials play an important role in the development of healthcare. However, the current status of clinical trials on anti-PD-1/PD-L1 for nasopharyngeal carcinoma remains unclear. Therefore, this study aims to provide a comprehensive analysis of the registered trials related to anti-PD-1/PD-L1 for nasopharyngeal carcinoma on ClinicalTrials.gov.

**Methods:** A search was conducted on the ClinicalTrials.gov database to identify all registered trials related to anti-PD-1/PD-L1 for nasopharyngeal carcinoma up to 26 February 2023. The characteristics of the trials were examined, and the studied drugs, disease conditions, as well as details of trials with available results were analyzed. Publication status was assessed by a PubMed search using the ClinicalTrials.gov NCT number.

**Results:** A total of 112 interventional clinical trials registered between 2015 and 2023 were included. Of the trials, 90 were carried out in Asia, 72 were in phase 2, and 31 trials had either companies or universities as sponsors/collaborators. The sample sizes across the trials varied greatly, with a median of 71.5 participants per trial. The majority of trials were recruiting participants, with only 6 had posted results. PD-1 inhibitors were preferred over PD-L1, and Toripalimab emerged as the most extensively studied drug. About one-third (33.9%) of the studies looked into recurrent/metastatic nasopharyngeal cancer.

**Conclusion:** This study provides an overview of all registered trials of anti-PD-1/PD-L1 for NPC. It is needed to improve the completeness, outcome selection, randomization and masking of trials and to be transparent and timely in reporting of results.

## Introduction

Nasopharyngeal carcinoma (NPC) is a type of epithelial carcinoma that arises from the mucosal lining of the nasopharynx (Chin et al., 2016). Compared to other malignancies, it is relatively infrequent. According to the report of the International Agency for Research on Cancer, there were 133,354 new cases of NPC worldwide in 2020, representing about 0.7% of all newly diagnosed cancers (Sung et al., 2021). The incidence of NPC is significantly unbalanced worldwide, with a significantly higher prevalence observed in East and Southeast Asia, accounting for over 70% of new cases (Chen et al., 2019). Therefore, standardized and comprehensive treatment of NPC is particularly important in high-risk areas and populations.

With the implementation of intensity-modulated radiotherapy (IMRT), a precision radiotherapy technique enabling conformation of high doses to concave-shaped tumors while protecting normal tissue, excellent loco-regional control rates have been achieved in the treatment of NPC(Lee et al., 2019; Killock, 2023). However, despite of patients diagnosed at early stages (stages I and II) with a favorable long-term survival, the majority of patients (∼80%) diagnosed at later stages due to the lack of early symptom, only have a 5-year survival rate of 70%–80% after appropriate therapy (Zhang et al., 2022). Most treatment failures, including recurrences and distant metastases, occur within 1–2 years after IMRT, indicating that a proportion of NPC may be radioresistant (Liu et al., 2017), suggesting that additional treatment approaches are needed for NPC.

Triggered by promising advances in immunotherapy, there has been growing interest in the use of immune checkpoint inhibitors (ICI), specifically anti-programmed death-1 or programmed death-1 ligand (PD-1/PD-L1) therapies in the treatment of NPC. Activated T lymphocytes express immune checkpoints such as PD-1 on the surface, which, when bound to ligands, transmit a “stop” signal to T cells and suppress the anti-tumor immune response (Pardoll, 2012). When tumor cells evade T-cell-mediated immune killing by over-expressing programmed cell death protein ligand 1 (PD-L1), they form immune escape by binding to immune checkpoints to disengage the receptor-ligand interaction between tumor cells and T cells (Renkvist et al., 2001). PD-1/PD-L1 therapies allows T cells to be effectively activated, thus restoring the body’s immune function to achieve anti-tumor effects, which is also believed to be a radiation enhancement factor (Wang et al., 2019; Vanneste et al., 2020). Being the commonest histological cell type, non-keratinizing NPC and is almost always associated with Epstein-Barr virus (EBV) infection (Lee et al., 2021). Non-keratinizing EBV^+^ NPC is characterized by a higher PD-L1 expression level and a pronounced lymphocytic infiltration in the tumor cell culture, rendering it a promising target for immunotherapy (Larbcharoensub et al., 2018; Outh-Gauer et al., 2018; Le et al., 2019). The approval of PD-1 inhibitors toripalimab and camrelizumab has facilitated the establishment of a standard treatment approach for patients with refractory recurrent/metastatic NPC (Xu et al., 2022).

Clinical trials play a vital role in evidence-based medicine and have been instrumental in driving the development of healthcare (Stensland et al., 2022). To ensure transparency in the clinical trial process, the International Committee of Medical Journal Editors (ICMJE) reached a consensus in 2004 that all clinical trials should be registered in a public registry before recruiting patients (De Angelis et al., 2004). This led to the establishment of ClinicalTrials.gov by the U.S. National Library of Medicine and the U.S. Food and Drug Administration in 2005, which currently holds over 446,966 research studies across 221 countries (https://clinicaltrials.gov/ct2/resources/trends). Consequently, it is considered as one of the most reliable sources of information for clinical trials. Accessing the information on ClinicalTrials.gov is expected to provide valuable insights into the current state of research and potential areas for further analysis. Despite previous studies conducted in other fields (Chen et al., 2018; Wang et al., 2020; Riddell et al., 2021), the status of registered trials of anti-PD-1/PD-L1 for NPC remains unknown.

Therefore, to gain a comprehensive overview of current clinical research progression on anti-PD-1/PD-L1 therapies for NPC, the study intends to undertake a thorough analysis of trials registered on ClinicalTrials.gov. The analysis focused on the current landscape of clinical trials on the use of anti-PD-1/PD-L1 therapies in NPC, including characteristics of trials and the study design, the distribution of drugs, as well as the disease conditions being targeted. By synthesizing this information, it is to provide a valuable resource for researchers, clinicians, and patients interested in the latest developments in the field of NPC immunotherapy.

## Materials and methods

### Data source and search strategy

A cross-sectional study was conducted to analyze clinical trials on anti-PD-1/PD-L1 for nasopharyngeal carcinoma (NPC) registered on ClinicalTrials.gov up to 26 February 2023. The trials were obtained using the advanced search function with the search term nasopharyngeal carcinoma OR NPC for “condition,” and PD-1 OR PD-L1 OR pembrolizumab OR nivolumab OR dostarlimab OR atezolizumab OR avelumab OR durvalumab OR toripalimab OR tislelizumab OR camrelizumab OR cemiplimab OR spartalizumab OR sintilimab OR sugemalimab or penpulimab for “intervention” with results limited to “interventional studies” (Vaddepally et al., 2020). Publication status was then assessed by a PubMed search and oncology conferences search, including ASCO, AACR, ESMO, and SITC. All the ClinicalTrials.gov NCT number were examined, and reports of the trials were analyzed.

### Inclusion and exclusion criteria

The inclusion criteria of the registered trials were clinical trials on anti-PD-1/PD-L1 therapies for NPC. To ensure the validity and accuracy of the analysis, trials meeting any of the following criteria were excluded: 1) trials involving patients with various solid tumors without separate data for the NPC patients; 2) trials lacked critical information or had incomplete data; or 3) trials with a non-interventional design that did not involve the administration of anti-PD-1/PD-L1 therapies.

### Data extraction and statistical analysis

A data extraction form was built to keep a record of the main characteristics of included trials. The following information was extracted: NCT number, title, status, study results, conditions, interventions, primary outcome measures, gender, age, phases, enrollment, funders, study type, allocation, intervention model, masking, start date, primary completion date, completion date, locations, etc. Descriptive statistics were utilized to characterize each trial, and categorical data were presented as frequencies and percentages.

Between-group comparisons for study design elements were performed using the Pearson χ2 test or the Fisher exact test if the number of studies in any single category was less than 10. Statistical analyses were completed on 20 June 2023, using SPSS Statistics Subscription Build, version 1.0.0.1461 (IBM Corp). Statistical significance was set at 2-sided *p* < .05. Since this study was based on publicly available data, ethical approval was not required.

## Results

### The characteristics of included trials

**FIGURE 1:**
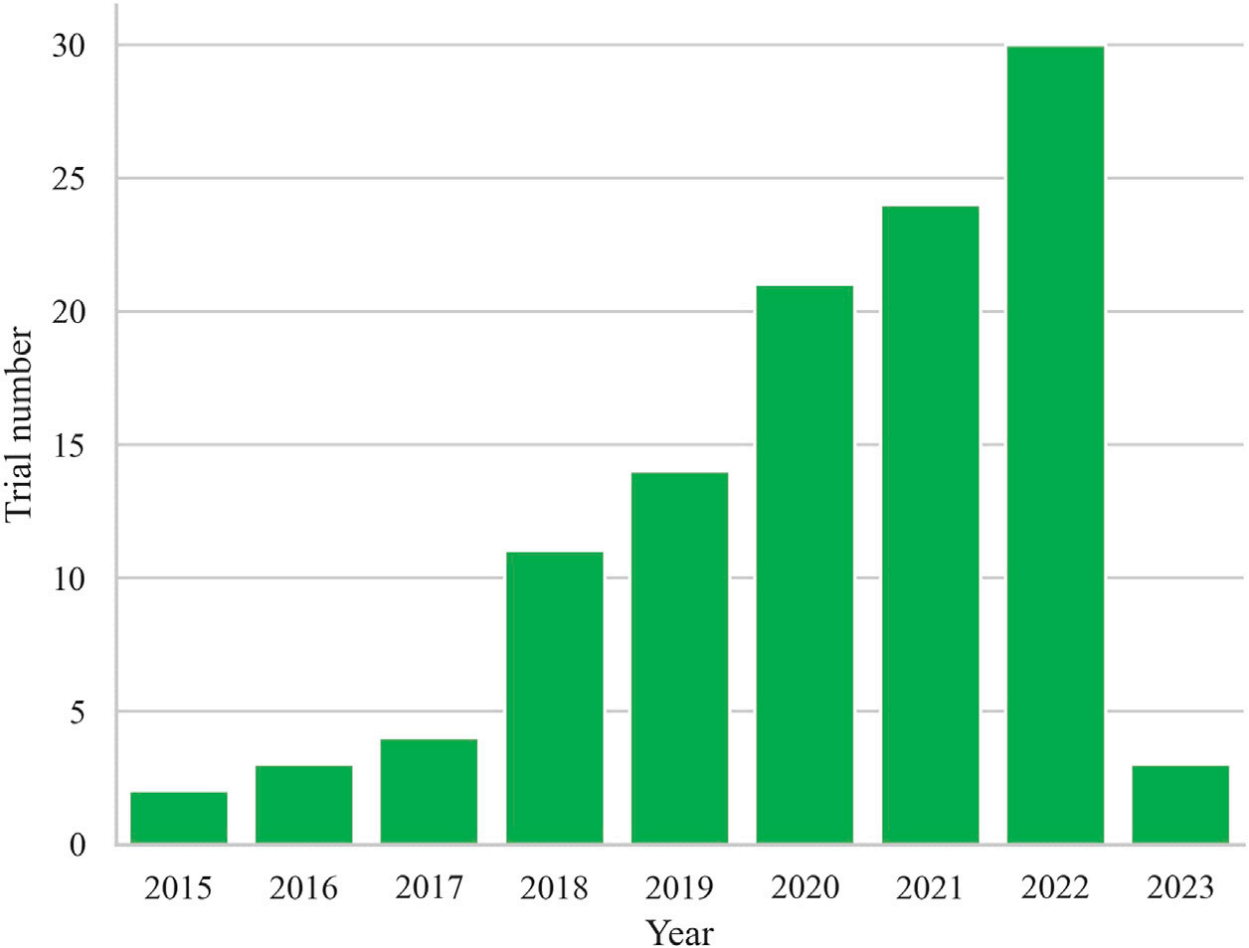
Annual trends of the number of registered trials.

Finally, a total of 112 registered trials were included. Table 1 shows the basic characteristics of the included 112 clinical trials. The annual trends of the number of registered trials, as depicted in Figure 1, reveal a steady increase since 2015, reaching their peak with the highest number of trials registered in 2022 (*n* = 30, 26.8%). Of the included trials, 51 (45.5%) were currently recruiting participants, while 24 (21.4%) had not yet started recruitment, and 22 (19.6%) were active but not recruiting. A small number of trials had completed (*n* = 8, 7.1%), terminated (*n* = 3, 2.7%), withdrawn (*n* = 2, 1.8%), or had an unknown status (n = 2, 1.8%). The majority of trials did not have available results (*n* = 106, 94.6%), while only 6 trials (5.4%) had available results.

**TABLE 1:** Characteristics of all included clinical trials.

Characteristics		Number	Percentage (%)
Start Year			
	2015	2	1.8
	2016	3	2.7
	2017	4	3.6
	2018	11	9.8
	2019	14	12.5
	2020	21	18.8
	2021	24	21.4
	2022	30	26.8
	2023	3	2.7
Status			
	Recruiting	51	45.5
	Not yet recruiting	24	21.4
	Active, not recruiting	22	19.6
	Completed	8	7.1
	Terminated	3	2.7
	Unknown status	2	1.8
	Withdrawn	2	1.8
Study results			
	No Results Available	106	94.6
	Has Results	6	5.4
Participant age			
	Adult, Older Adult	109	97.3
	Child, Adult, Older Adult	3	2.7
		
	≤50	52	46.4
	51–100	15	13.4
	101–500	43	38.4
	≥501	2	1.8
Location			
	Asia	90	80.4
	North America	10	8.9
	Global	10	8.9
	Europe	2	1.8
Type of sponsor/collaborators			
	Company	31	27.7
	University	31	27.7
	Affiliated Hospital	19	17.0
	University and Affiliated Hospital	15	13.4
	University and Company	8	7.1
	National institute	4	3.6
	University and National Institute	2	1.8
	Affiliated Hospital and Company	1	0.9
	Affiliated Hospital and National institute	1	0.9
Number of sponsor and collaborators			
	1	65	58.0
	2–5	34	30.4
	6–10	10	8.9
	11–15	3	2.7
Number of sites/centers			
	1	44	39.3
	2–5	9	8.0
	6–10	8	7.1
	11–20	8	7.1
	21–50	8	7.1
	>50	2	1.8
	Not provided	35	31.3
Funded by			
	Other	73	65.2
	Industry	22	19.6
	Industry and Other	12	10.7
	NIH and Other	3	2.7
	NIH	2	1.8

Abbreviation: NIH, national institutes of health.

In terms of participant demographics, most trials included only adult and older adult participants (n = 109, 97.3%), with only a few trials including children participants (*n* = 3, 2.7%). Most trials would enroll 101–500 participants (*n* = 43, 38.4%), while 52 trials (46.4%) would enroll ≤ 50 participants, and 15 trials (13.4%) would enroll 51–100 participants. Only 2 trials would enroll ≥ 501 participants. Geographically, most trials would conduct in Asian (*n* = 90, 80.4%), followed by North America (*n* = 10, 8.9%), Global (*n* = 10, 8.9%), Europe (*n* = 2, 1.8%).

It appears that the most common sponsors/collaborators were companies and universities alone, each comprising 31 trials (27.7%). Hospitals were the third most common sponsor/collaborator, with 19 trials (17% of the trials). Interestingly, a significant portion of the trials (*n* = 15, 13.4%) had both a university and hospital as sponsors/collaborators. Regarding the number of sponsors and collaborators, the majority of trials (*n* = 65, 58%) had only one sponsor or collaborator, while 34 trials (30.4%) had between 2–5 sponsors or collaborators. A smaller proportion of trials had between 6–10 (*n* = 10, 8.9%) or 11–15 (*n* = 3, 2.7%) sponsors/collaborators.

Among the trials included in the analysis, 44 trials (39.3% of the total) were conducted at a single site/center. 9 trials (8.0%) were conducted at 2 to 5 sites/centers, 8 trials (7.1%) were conducted at 6 to 10 sites/centers, 8 trials (7.1%) were conducted at 11 to 20 sites/centers, and 8 trials (7.1%) were conducted at 21 to 50 sites/centers. There were 2 trials (1.8%) that involved more than 50 sites/centers. The information regarding the sites/centers was not provided for 35 trials (31.3%).

**FIGURE 2:**
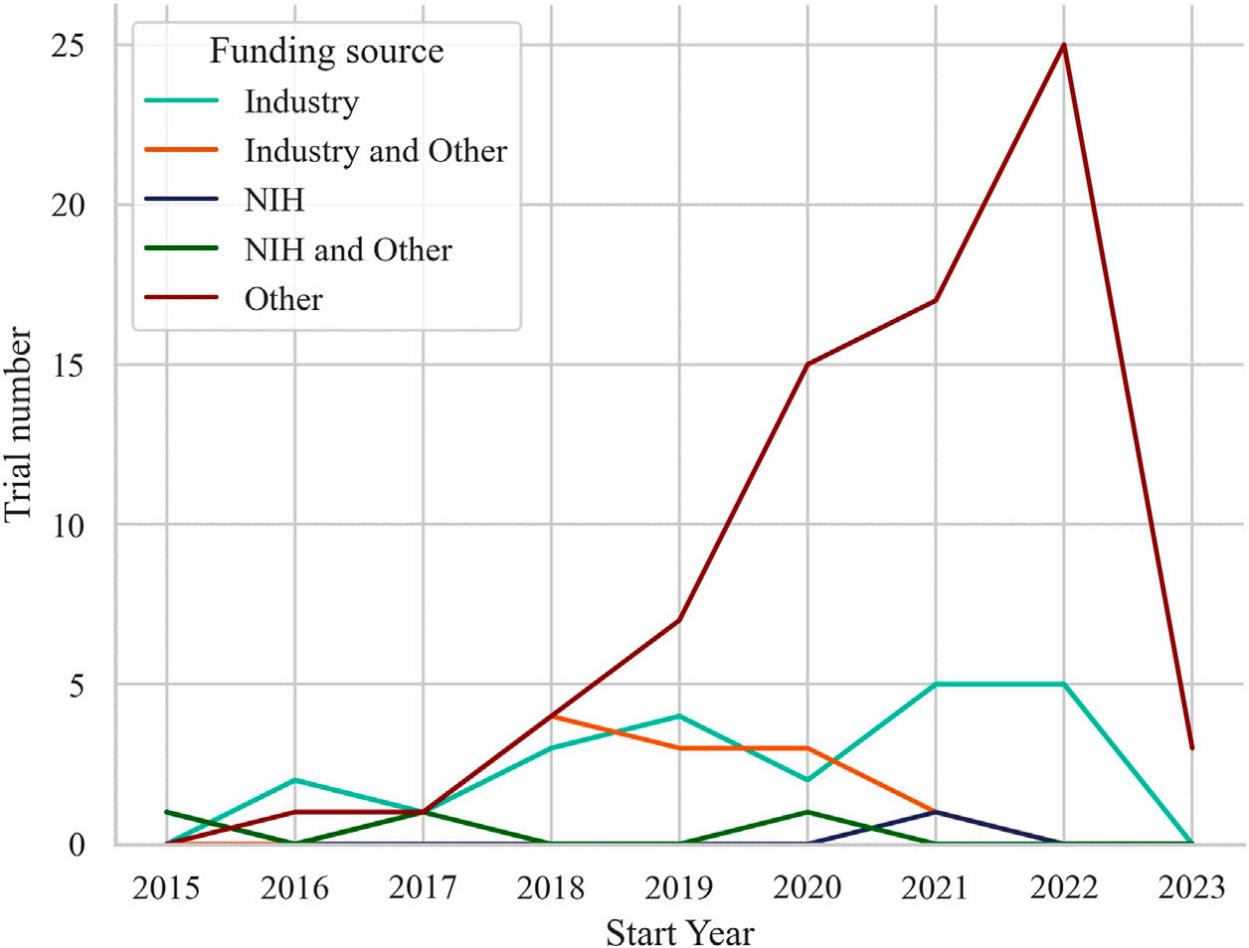
Number of trials per year based on their funding source.

Funding for the trials was primarily from other sources (*n* = 73, 65.2%), with 22 trials (19.6%) funded by industry, 12 trials (10.7%) funded by industry and other, and 3 trials (2.7%) funded by National Institutes of Health (NIH) and other. Only 2 trials (1.8%) were funded by NIH alone. Annually, the distribution of trials also varied across different funding sources as displayed in Figure 2. Other-funded trials raised along with the trend of all trials, from 1 trial in 2016 to 25 trials in 2022. Industry-funded trials showed fluctuating numbers over the years, ranging from 2 trials in 2016 to 5 trials in both 2021 and 2022. Trials funded by industry and other sources had 1 trial in 2017, increasing to 4 trials in 2018, and then ranging from 1 to 3 trials in subsequent years. The 3 trials received funding from both the NIH and other sources distributed across 2015, 2017, and 2020, and the 2 trials solely funded by the NIH distributed with one in 2015 and the other in 2021.

### Characteristics of study design

Study design characteristics of included trials are displayed in Table 2. Among the 112 trials, phase 2 trials were the most common (*n* = 72, 64.3%), followed by phase 3 trials (*n* = 18, 16.1%), and phase 1 trials (*n* = 12, 10.7%). In terms of allocation, 40 trials (35.7%) were randomized, 15 (13.4%) were non-randomized, and 57 (50.9%) did not report allocation information. Single-group assignment was the most common intervention model (*n* = 58, 51.8%), followed by parallel assignment (*n* = 49, 43.8%), and sequential assignment (*n* = 4, 3.6%). The majority of trials (*n* = 103, 92%) had no masking, while a small number of trials had single (*n* = 2, 1.8%), double (*n* = 3, 2.7%), or quadruple (*n* = 4, 3.6%) masking. Among the 112 trials, phase 2 trials were the most common (*n* = 72, 64.3%), followed by phase 3 trials (*n* = 18, 16.1%), and phase 1 trials (*n* = 12, 10.7%). In terms of allocation, 40 trials (35.7%) were randomized, 15 (13.4%) were non-randomized, and 57 (50.9%) did not report allocation information. Single-group assignment was the most common intervention model (*n* = 58, 51.8%), followed by parallel assignment (*n* = 49, 43.8%), and sequential assignment (*n* = 4, 3.6%). The type of primary outcomes varied, with response rate (Objective response rate (ORR), best overall response rate (BORR), and complete response (CR)) and survival data other than overall survival (progress-free survival (PFS), prolong one-year disease free survival (DFS-1y), failure-free survival (FFS), disease-free survival, etc.) being the most common outcomes (37.5% and 36.6%, respectively). Toxicity or safety/tolerability were also primary outcomes for a considerable proportion of trials (*n* = 22, 19.6%), and only 7 trials (6.3%) set OS as the primary outcome.

**TABLE 2:** Characteristics of study design.

Characteristics		Number	Percentage (%)
Phases			
	Phase 1	12	10.7
	Phase 1|Phase 2	9	8.0
	Phase 2	72	64.3
	Phase 2|Phase 3	1	0.9
	Phase 3	18	16.1
Allocation			
	Non-Randomized	15	13.4
	Randomized	40	35.7
	N/A	57	50.9
Intervention model			
	Factorial Assignment	1	0.9
	Parallel Assignment	49	43.8
	Sequential Assignment	4	3.6
	Single Group Assignment	58	51.8
Masking in randomized studies			
	None	31	77.5
	Single	2	5.0
	Double	3	7.5
	Quadruple	4	10.0
Type of Primary Outcome			
	Response Rate	42	37.5
	Other Survival Data	41	36.6
	Toxicity or Safety	22	19.6
	Overall Survival	7	6.3

Abbreviation: N/A, not applicable.

Table 3 presents several key trial characteristics observed across phases of clinical research. In terms of blinding, Phase 3 trials exhibited blinding in 33.3% of cases, contrasting with Phase 2 trials where blinding was employed in only 4.2% of studies; conspicuously, blinding was entirely absent in Phase 1 trials. The median number of participants in these trials displayed notable variations: Phase 3 trials featured a median of 276 participants, encompassing a range of 200–556, while Phase 2 trials had a median of 47 participants, spanning a range of 14–206. Phase 1 trials, on the other hand, had a median of 108.5 participants. The participant numbers in the Phase 1 trials included in the study exhibited significant variation, ranging from 23 to 242, owing to the inclusion of various solid tumors. As for multicenter involvement, Phase 3 trials were notably prevalent in this regard, with 83.3% of trials engaging multiple centers, in contrast to Phase 2 where only 33.3% of trials embraced multicenter collaboration; Phase 1 trials exhibited intermediate multicenter participation, accounting for 58.3% of the trials. In terms of the utilization of randomization, Phase 3 trials consistently employed randomization in all cases, totaling 100% implementation, reflecting a rigorous allocation approach. Phase 2 trials showed a more modest utilization of randomization at 25.0%, while none of the Phase 1 trials employed randomization. For outcome measures, overwhelmingly more Phase 1 trials focused on safety than other phases, while more Phase 3 trials set OS as their primary outcome.

**TABLE 3:** Comparison of randomized and non-randomized trials.

Characteristic	Trial phase		
	Phase 3 (*n* = 18)	Phase 2 (*n* = 72)	Phase 1 (*n* = 12)
Blinding (%)	6 (33.3)	3 (4.2)	0 (0)
No. of participants, median (Range)	276 (200–556)	47 (14–206)	108.5 (23–242)
Multicenter (%)	15 (83.3)	24 (33.3)	7 (58.3)
Randomization (%)	18 (100)	18 (25.0)	0 (0)
Primary outcome as safety (%)	0 (0)	4 (5.6)	7 (58.3)
Primary outcome as OS(%)	5 (27.8)	2 (2.8)	0 (0)

Abbreviation: OS, overall survival.

### Overview of drugs

Table 4 shows the types and number of drugs used in clinical trials targeting the PD-1 and PD-L1 pathways. PD-1 inhibitors were the most studied drugs, with Toripalimab being used in the highest number of trials (*n* = 25), followed by Camrelizumab (*n* = 13), Pembrolizumab (*n* = 13), Sintilimab (*n* = 9), Tislelizumab (*n* = 9), and Nivolumab (*n* = 7), while Penpulimab, Spartalizumab, and Serplulimab were used in a small number of trials. Among PD-L1 inhibitors, Avelumab was used in the highest number of trials (3), followed by Atezolizumab, Durvalumab, SHR-1701, Envafolimab, INCB099280, INCB099318, TBQ2450, and TQB2858, each used in 1–2 trials. It is worthy to know that the Cadonilimab, a PD-1/CTLA-4 bi-specific antibody, has been analyzed in 2 clinical trials for the treatment of NPC.

**TABLE 4:** Types, names and trial attribution of drugs.

Types	Drug names	Trial number
PD-1		
	Toripalimab	25
	PD-1 inhibitor	14
	Camrelizumab	13
	Pembrolizumab	13
	Sintilimab	9
	Tislelizumab	9
	Nivolumab	7
	Penpulimab	3
	Spartalizumab	2
	Serplulimab	1
PD-L1		
	Avelumab	3
	Atezolizumab	2
	Durvalumab	2
	SHR-1701	2
	Envafolimab	1
	INCB099280	1
	INCB099318	1
	TBQ2450	1
	TQB2858	1
PD-1/CTLA-4		
	Cadonilimab	2

In the top five most studied PD-1 inhibitors, four were developed by Chinese pharmaceutical companies, with Toripalimab by Junshi Biosciences being used in the highest number of trials, followed by Camrelizumab by Jiangsu Hengrui Medicine, Sintilimab by Innovent Biologics, and Tislelizumab by BeiGene. The only non-Chinese PD-1 inhibitor in the top five was Pembrolizumab, developed by Merck & Co., Inc.

### Overview of NPC conditions

Table 5 displays the distribution of different conditions in the included interventional trials. The most common condition studied was recurrent/metastatic nasopharyngeal cancer, with 38 trials (33.9%) of this condition. This was followed by locoregionally advanced nasopharyngeal carcinoma (26.8%). Other conditions included metastatic nasopharyngeal carcinoma (11.6%), advanced nasopharyngeal carcinoma (11.6%), recurrent nasopharyngeal carcinoma (8.9%). Lastly, 3 (2.7%) trials were conducted on EBV^+^ NPC, and 1 (0.9%) each on stage II-IVB nasopharyngeal carcinoma and progression during or after platinum-based treatment.

**TABLE 5:** The details of NPC Conditions.

Conditions	Numbers	Percentage
Recurrent/Metastatic Nasopharyngeal Carcinoma	38	33.9
Locoregionally Advanced Nasopharyngeal Carcinoma	30	26.8
Metastatic Nasopharyngeal Carcinoma	13	11.6
Advanced Nasopharyngeal Carcinoma	13	11.6
Recurrent Nasopharyngeal Carcinoma	10	8.9
EBV^+^ NPC	3	2.7
High-risk Nasopharyngeal Carcinoma	3	2.7
Progression During or After Platinum-based Treatment	1	0.9
Stage II-IVB Nasopharyngeal Carcinoma	1	0.9

Abbreviation: EBV^+^. NPC, Epstein-Barr Virus-associated nasopharyngeal carcinoma.

### Characteristics of trials with results available on ClinicalTrials.gov

Table 6 provides the information on 6 clinical trials with available results, including the drug name, phase of the trial, disease condition studied, funding source, and sponsor/collaborators. Notably, 5 out of the 6 trials analyzed PD-1 inhibitors (Avelumab, Nivolumab, Pembrolizumab, and Toripalimab), while the remaining one analyzed a PD-L1 inhibitor (Atezolizumab). Of the 5 PD-1 inhibitor trials, 3 were phase 2 trials (Avelumab, Nivolumab, and Spartalizumab), while the remaining 2 were phase 3 trials (Pembrolizumab and Toripalimab). Among these trials, only the Nivolumab phase 2 trial was funded by the NIH, while the other 4 trials were funded by industry and other sources. It is also worth noting that all 5 trials targeted recurrent/metastatic NPC. For Pembrolizumab, both phase 1/2 and phase 3 trials were registered, with the phase 1/2 trial being funded by the NIH and other sources and the phase 3 trial being funded by Merck Sharp & Dohme LLC.

**TABLE 6:** The details of trials with available results.

NCT number	Name	Phase of trial	Disease	Funded by	Sponsor/Collaborators
NCT02875613	Avelumab	2	R/M	Industry and Other	Assuntina Sacco, M.D.|Pfizer|University of California, San Diego
NCT02605967	Spartalizumab	2	R/M	Industry	Novartis Pharmaceuticals|Novartis
NCT02339558	Nivolumab	2	R/M	NIH	National Cancer Institute (NCI)
NCT02611960	Pembrolizumab	3	R/M	Industry	Merck Sharp & Dohme LLC
NCT03581786	Toripalimab	3	R/M	Other	Shanghai Junshi Bioscience Co., Ltd.
NCT02538510	Pembrolizumab	1|2	R/M	NIH and Other	University of Washington|NCI

Abbreviation: R/M, Recurrent/metastatic nasopharyngeal carcinoma; NIH, national institutes of health. Characteristics of trials with results published on PubMed and major oncology conferences.

Table 7 provides the information on 12 clinical trials with available results on PubMed and 5 trials with results presented on major oncology conferences, including the drug name, phase of the trial, disease condition studied, whether the primary end point was positive or not, and the impact factor (IF) of the journal according to Journal Citation Reports Social Sciences Edition (Clarivate Analytics, 2023) in which the studies were published, or the name of the posted conference. It can be noticed that all trials focused on late-stage, especially R/M NPC. Two phase 3 trials, NCT03581786 and NCT03707509 both met the primary endpoint, and were published on journals with highest IF, reaching 82.9 and 51.1 respectively. In contrast, trials that did not met positive findings on the primary end point (NCT02605967 and NCT03097939) were published on journals with relatively lower IF of 11.5 and 16.6.

**TABLE 7:** The details of trials with available results.

NCT number	Drug name	Phase	Specific conditions	Primary end point	IF/CN
NCT04073784	Toripalimab	Phase 1	Locoregionally Advanced	N/A	17.0
NCT02605967	Spartalizumab	Phase 2	R/M	Negative	11.5
NCT03097939	Nivolumab and Ipilimumab	Phase 2	R/M	Negative	16.6
NCT02339558	Nivolumab	Phase 2	R/M	N/A	45.3
NCT03924986	Tislelizumab	Phase 3	R/M	Positive	50.3
NCT03854838	Toripalimab	Phase 2	R	N/A	10.9
NCT02915432	Toripalimab	Phase 1|Phase 2	R/M	N/A	45.3
NCT03581786	Toripalimab	Phase 3	R/M	Positive	82.9
NCT02538510	Pembrolizumab	Phase 1|Phase 2	R/M	N/A	11.5
NCT03074513	Atezolizumab	Phase 2	Stage IV AJCC v7	N/A	28.4
NCT03707509	Camrelizumab	Phase 3	R/M	Positive	51.1
NCT03121716	Camrelizumab	Phase 1	R/M	N/A	51.1
NCT05448885	Tislelizumab	Phase 2	Locoregionally Advanced	N/A	ESMO
NCT05166577	Pembrolizumab	Phase 1|Phase 2	R/M	N/A	ESMO
NCT05549466	Camrelizumab	Phase 2	R/M	N/A	ASCO
NCT03866967	Penpulimab	Phase 2	M	Positive	ESMO
NCT03558191	Camrelizumab	Phase 2	R/M	Positive	ESMO

Abbreviation: R/M, Recurrent/metastatic nasopharyngeal carcinoma; N/A, not applicable; IF, impact factor; CN, conference name; AJCC, the american joint committee on cancer.

### Characteristics of early discontinued trials

Table 8 provides an overview of the trials included in the analysis that experienced early discontinuation. The phase 2 trial registered under NCT02875613, which began in January 2017, was terminated due to slow patient accrual. It had an enrollment of 6 participants. Similarly, the phase 1/phase 2 trial registered under NCT03769467, which started in February 2019, was terminated with an enrollment of 12 participants for the difficulty in recruiting patients. The phase 2 trial registered under NCT03544099, initiated in May 2019, was terminated because the sponsor withdrew funding. Only 2 participants were enrolled in this trial. On the other hand, two phase 2 trials, NCT03390738 and NCT04231864, were withdrawn without enrolling any participants. The reasons stated for withdrawal were sponsor termination and feasibility concerns respectively.

**TABLE 8:** The details of trials with early discontinuation.

NCT number	Start date	Status	Phases	Enrollment	Reason
NCT02875613	Jan 2017	Terminated	Phase 2	6	Slow patient accrual
NCT03544099	May 2019	Terminated	Phase 2	2	Sponsor withdrew funding
NCT03769467	Feb 2019	Terminated	Phase 1|Phase 2	12	Difficult to recruit Patients
NCT03390738	June 2018	Withdrawn	Phase 2	0	Sponsor decided to terminate
NCT04231864	Dec 2020	Withdrawn	Phase 2	0	Feasibility

### Diverse treatment approaches across different lines of therapy

**FIGURE 3:**
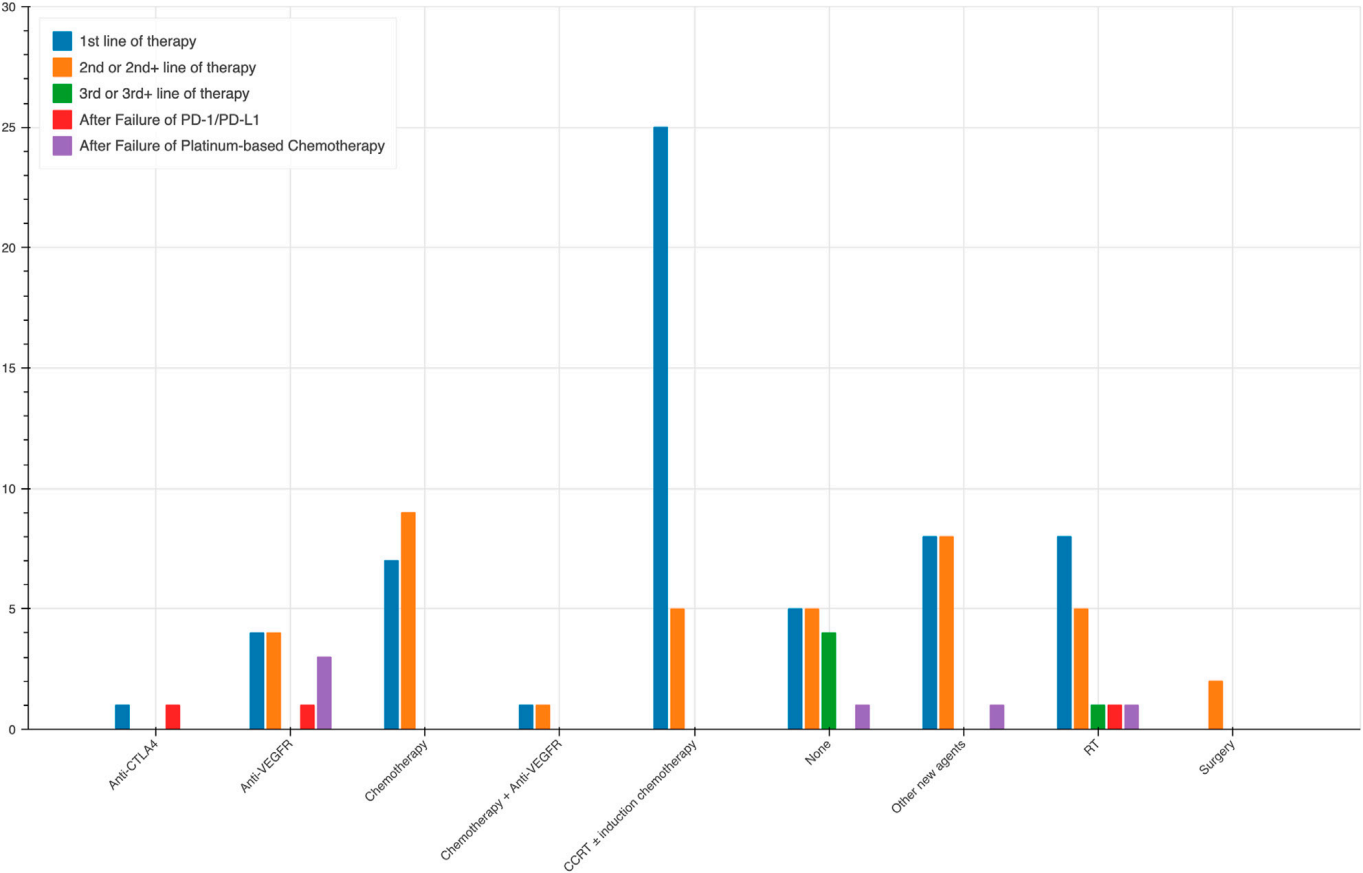
Different combination across lines of therapy.

During our analysis, we observed notable trends in the selection of treatment modalities based on the lines of therapy. Apart from 15 trials using PD-1/PD-L1 monotherapy, the rest 97 trials combined it with other agents. The different combination across lines of therapy are shown in Figure 3. In trials that adopted anti-PD-1/PD-L1 as the first line of therapy, induction chemotherapy combined with radiotherapy was the predominant choice, employed in 25 trials, highlighting its significance as an initial strategy in NPC treatment. Furthermore, chemotherapy alone was utilized in 7 trials, while a combination of chemotherapy and anti-VEGFR therapy was employed in 1 trial. Radiation therapy (RT) was chosen in eight trials, and surgery in eight trials. Additionally, one trial each utilized anti-CTLA4 therapy, anti-VEGFR therapy, and other new agents along with anti-PD-1/PD-L1 as the first line of treatment. In the second or second + lines of therapy, CCRT without induction chemotherapy was still a commonly utilized combination, albeit less frequently, being employed in 5 trials. Chemotherapy alone was used in 9 trials, anti-VEGFR therapy alone was opted for 4 trials, while a combination of chemotherapy and anti-VEGFR therapy was employed in one trial. In this context, RT was chosen in five trials, surgery in two trials, and five trials did not involve any other specific treatment.

For the third or third + lines of therapy, the data is limited, with only one trial incorporating RT along with PD-1/PD-L1 therapy. In the post anti-PD-1/PD-L1 and post Platinum-based Chemotherapy settings, the use of anti-VEGFR therapy was more common, being employed in three trials and one trial, respectively. Additionally, single trials in each of these settings utilized chemotherapy and anti-CTLA4 therapy.

## Discussion

This study provides an overview of the registered trials on ClinicalTrials.gov regarding anti-PD-1/PD-L1 therapy for NPC. The registered trials increased steadily from 2015 to 2022, with most being conducted in Asia. Only a small number of trials were funded by the NIH, while most of the trials received funding from sources other than the NIH.

Many trials included in this study were either recruiting or not yet recruiting and enrolled between 101 and 500 participants. Phase 2 trials were the most used but also the most withdrawn or terminated study design, and single-group assignment was the most frequently employed intervention. It is demonstrated that current trials on anti-PD-1/PD-L1 therapy for NPC were predominantly incomplete, early-phase studies with a generally high proportion of single-group assignment studies. These findings indicated that the current data of anti-PD-1/PD-L1 immunotherapy for NPC was largely in early-stage discovery. It is worth noting that all 18 phase 3 trials included in the analysis adopted randomization and parallel assignment, with an average enrollment of 325 participants. However, only 2 trials used quadruple masking (participant, care provider, investigator, and outcomes assessor), 1 used double masking (participant and investigator), and 2 used single masking (outcomes assessor), while the remaining 13 trials were open-labelled. Randomization is a crucial aspect of high-quality clinical trials as it ensures that the treatment is received by a certain proportion of patients, and that the treatment groups being compared are comparable in both measured and unmeasured patient characteristics (Bespalov et al., 2020). By minimizing bias and increasing the reliability of evidence, randomization has become a hallmark of high-quality clinical trials (Kunz et al., 2007). Therefore, it is important for researchers to consider adopting randomization and blinding whenever feasible (Broglio, 2018).

Most trials used response rate (mainly ORR) and survival data other than OS (mainly PFS) the primary outcomes. ORR is a widely accepted endpoint in single-arm trials as it allows for measurable tumor response without requiring a control group for direct comparison. PFS, on the other hand, is defined as the time from the start of therapy to the first documented tumor progression or death due to any cause (Kemp and Prasad, 2017). The measurement of PFS is complex, and bias can be introduced in PFS assessment depending on the adequacy of the comparator used. In comparison, OS, defined as the time from treatment initiation to death, remains the gold-standard clinical endpoint for oncology cytotoxic clinical trials (Anagnostou et al., 2017). However, OS measurement can be resource-intensive and time-consuming. More recently, the majority of accelerated approvals have been based on ORR (Beaver et al., 2018; Batta et al., 2020); these approvals are conditional and require subsequent confirmation of benefit, such as PFS or OS, in larger and/or randomized studies.

Based on the analysis of multiple drugs, PD-1 inhibitors were found to have undergone the most extensive research, with Toripalimab being the most commonly utilized agent. Conversely, Avelumab was identified as the most frequently studied PD-L1 inhibitor. Whether anti-PD-1 and anti-PD-L1 deliver different clinical outcomes remains a topic of controversy, and systematic reviews and meta-analyses conducted yielded varying outcomes regarding the clinical performance of different immune check-point inhibitors through indirect comparison (Wu et al., 2018; You et al., 2018). The current study indicated that anti-PD-1 was more favorable by researchers and might demonstrate superior clinical outcomes in NPC patients compared to anti-PD-L1. Nonetheless, head-to-head studies were necessary for a direct comparison between alternative interventions.”

It is notable that among the top studied PD-1 inhibitors for NPC, the majority were developed by Chinese pharmaceutical companies. However, the trials initiated by the United States industry and NIH demonstrated a higher rate of result postings. This suggested a potential need for improvement in the conduct of clinical trials by Chinese universities and companies, particularly in terms of timely reporting of trials’ results. Future efforts should be aimed at ensuring that all trials, regardless of funding source or sponsor, were conducted in accordance with best practices for clinical research and that their results should be reported in a transparent and timely manner. Recurrent/metastatic nasopharyngeal cancer and locoregionally advanced nasopharyngeal carcinoma were the most studied conditions in the study. The finding is noteworthy as it aligns with the unmet clinical needs in the management of nasopharyngeal carcinoma (Le et al., 2019; Wong et al., 2021).

The choice of publication journal can significantly impact the visibility and influence of clinical trial findings. The findings from trials published in high-IF journals have a greater likelihood of influencing clinical practice and shaping future research directions (Evangelou et al., 2012). Our analysis suggests a potential correlation between the positive trial outcomes and the selection of high-IF journals for publication. On the other hand, trials with negative or inconclusive outcomes may face challenges in publication acceptance, particularly in high-IF journals. This phenomenon could stem from a publication bias favoring positive results or a higher threshold for acceptance in prestigious journals. It is important to acknowledge that the IF is just one factor in evaluating the quality and significance of research publications. Other considerations, such as study design, methodology, and scientific rigor, also contribute to the overall credibility and influence of a study.

The choice of treatment modalities in NPC management is influenced by the line of therapy, with induction chemotherapy combined with radiotherapy being notable options in the early lines of treatment, while anti-VEGFR therapy and other therapies become more relevant in later lines and post-treatment scenarios.

The study had several limitations that need to be acknowledged. Firstly, the scope of the analysis was restricted to clinical trials registered in ClinicalTrials.gov, which may not include all clinical trials, as some investigators or sponsors may register their studies in other databases (Yang et al., 2021). Secondly, the study only provided an overview of the registered trials’ characteristics and did not assess the actual strengths and weaknesses of the clinical studies. Thirdly, the search strategy might have missed trials that studied solid tumors if NPC was not mentioned in the inclusion criteria, such as the well-known KEYNOTE-028 and NCI-9742 studies (Ma et al., 2018; Ott et al., 2019). However, given the rarity of these types of large-scale trials, it is unlikely that the absence of these studies would significantly impact the conclusions drawn from the research. Additionally, the validity of the data in ClinicalTrials.gov was contingent on the quality of information provided by the sponsors, and missing data in certain fields might introduce bias into the results. These limitations should be taken into consideration when interpreting the findings.

In conclusion, the current study provides an overview of clinical trials investigating anti-PD-1/PD-L1 therapies for NPC. The analysis highlights the need for improvement in completeness, outcome selection, randomization and masking of trials.

## Data Availability

The original contributions presented in the study are included in the article/Supplementary Material, further inquiries can be directed to the corresponding author.
